# Death of a Close Relative and the Risk of Suicide in Sweden—A Large Scale Register-Based Case-Crossover Study

**DOI:** 10.1371/journal.pone.0164274

**Published:** 2016-10-11

**Authors:** Hanna Mogensen, Jette Möller, Hanna Hultin, Ellenor Mittendorfer-Rutz

**Affiliations:** 1 Karolinska Institutet, Department of Public Health Sciences, Stockholm, Sweden; 2 Karolinska Institutet, Institute of Environmental Medicine, Stockholm, Sweden; 3 Karolinska Institutet, Department of Clinical Neuroscience, Stockholm, Sweden; Medical University Vienna, AUSTRIA

## Abstract

**Background:**

Bereavement is thought to be a risk factor for suicide but the association has not been thoroughly investigated according to specific sensitive time periods and risk groups using a self-matched design. We aimed to 1) determine the risk of suicide within the first year after death of a close relative, 2) investigate if and how the risk changes within this time window and 3) determine if sex, age, and type of relationship, affect this association.

**Methods:**

A self-matched, case-crossover study was performed by linking Swedish registers. In total, 31 059 individuals with suicide between 1990 and 2011 were included. Different periods within the year prior to the suicide were compared with corresponding periods one year earlier in the same individual’s life. Conditional logistic regression was used to calculate odds ratios (OR) and 95% confidence intervals (CI) for suicide after death of a close relative.

**Results:**

Increased ORs of suicide were seen during the first month, OR 1·77 (95% CI 1·35–2·34), and the first half-year, 1·27 (1·13–1·43). An even higher OR was found within the first week, 3·43 (1·89–6·22). Patterns were similar for women and men and across age groups. Death of a partner or child but not death of a sibling or parent was associated with a significantly increased suicide risk. The strongest association was seen after death of a partner in individuals aged 45 and older.

**Discussion:**

These findings provide knowledge of sensitive time periods and at-risk groups in the early period of bereavement. Due to the use of a self-matched study design, methodological challenges of unmeasured residual confounding could be overcome.

## Introduction

The death of a close relative constitutes an exceptionally stressful life event [1,2]. The grieving process may include aspects of anger, guilt and despair and is often experienced in the context of social isolation. Studies investigating the burden of grief have reported an elevated risk of adverse health outcomes, including suicide after the loss [3–10]. In order to improve targeted support for bereaved individuals, identifying risk groups and specific sensitive time periods is crucial. The available literature suggests that pathways to negative consequences after bereavement may vary with sex and age as well as the cause (suicide or other causes) and the type (e.g. partner) of bereavement [1,4,5,7–10]. Still, to date the literature with regard to the risk of suicide related to the relation to the bereaved, particularly after loss of child, sibling or parent and with regard to the duration of bereavement is sparse. Reported time windows of excess suicide risk after bereavement include the first week, first month, first six months and first year [6–9].

The response to bereavement may vary individually with regard to the existence of health problems and psychosocial adversities prior to bereavement [1]. Due to this individual variation, it is important to have adequate information on a number of potential confounders in bereavement studies. To the best of our knowledge, this is the first study in this research field related to bereavement and subsequent suicide risk applying a self-matched study design in order to overcome the methodological challenges of unmeasured residual confounding. In a case-crossover design, every case acts as his/her own control, which implies that the design inherently controls for confounders that are constant over time within one individual, like e.g. sex and genetics [11].

The aims of this study were to: 1) determine the short-term risk of suicide within the first year after death of a close relative; 2) investigate if and how the risk changes within this time window; and 3) determine if sex and age of the bereaved, and type of relationship, affect this association.

## Materials and Methods

Information from three Swedish registers (the Total Population Register, the Multi-Generation Register and the Cause of Death Register) were linked by using unique, de-identified Swedish personal identity numbers. From the Cause of Death Register, all suicides between 1990 and 2011, N = 34 404 individuals aged 10 or older, were identified. Individuals with unknown date of death were excluded (n = 3 345, 9·7%), which left 31 059 individuals in the study population (index persons). No statistical difference with regard to sex and mean age was found between the index persons and the individuals excluded due to missing date of death.

Suicide was defined by the underlying cause of death or contributing cause of death. Both “certain” and “uncertain” suicides were included. A “certain” suicide was defined as death due to intentional self-harm using the International Classification of Diseases (ICD) codes for version 9 and 10 (ICD-9: E950-E959, ICD-10: X60-X84). An “uncertain” suicide was defined as an event of undetermined intent (ICD-9: E980-E989, ICD-10: Y10-Y34). By inclusion of “certain” and “uncertain” suicides, the study takes geographical and temporal changes in ascertainment methods and the frequent underreporting of suicide into consideration. This procedure is often preferred in this research field [12,13]. To explore the accuracy of combining “certain” and “uncertain” suicides, we performed a sensitivity analysis including only “certain” suicides (n = 24 176, 77·8%) with regard to exposure to any close relative’s death. The analysis revealed the comparability of the combined estimates with the estimates for “certain” suicide.

### Case-crossover design

We employed a case-crossover design, in which the study population consisted of cases and each case served as his/her own control [11]. The study design resembles a matched case-control study, but instead of comparing different individuals at the same point in time, each individual is compared with him/herself at different points in time, defined as case and control periods [11]. The case-crossover design is purposeful for studying exposures with a transient effect on an acute outcome, and has previously been used to study e.g. the risk of suicide in relation to diagnosis of cancer [11,14].

### Exposure

The exposure was defined as the death of a parent (biological or adoptive), sibling (biological), child (biological or adopted), or partner (spouse, registered same-sex partner, or opposite-sex cohabitants with common child). Linkage with the Multi-Generation Register, covering index persons born 1932 and after (n = 24 452, 78·7%), supplied information on parents, children, and siblings [15]. Information on partners was linked using the Total Population Register. Due to changes in registration systems, information regarding partners could only be obtained for index persons with a death date occurring on 1^st^ January 1993 or later (n = 25 985, 83·7%). Furthermore, only partners that could be linked to the index persons during the two calendar years prior to the years of the analysed time periods (i.e. case/control periods), were considered in order to restrict the analyses to current or recent partners.

Information regarding the relatives’ date of death was obtained from the Cause of Death Register. Deaths of relatives occurring between 1988 and 2011 were included as exposure events. Exposure was defined as having at least one close relative who died within the analysed periods. Relatives with a missing date of death (<2%) were not included.

### Statistical analysis

**Fig 1:**
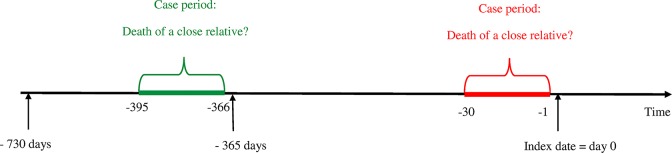
Graphical visualization of case and control periods when the first month preceding the suicide was used as case period.

The odds of being exposed to a close relative’s death in the year (i.e. 1–365 days) before the suicide (case period) was compared with the odds of being exposed in the year before that (i.e. 366–730 days preceding the index person’s suicide, control period). To estimate if and how the relative risk of suicide changes within the first year of bereavement, different case periods within this year were analysed separately; the first and the second half-year, the twelve months and the four weeks preceding the suicide. The comparisons were made with the corresponding time periods in the control period. See illustration in Fig 1.

Odds ratios (OR) and corresponding 95% confidence intervals (CI) were calculated using conditional logistic regression. The calculated OR of exposure can be interpreted as an estimation of the relative risk of suicide after death of a close relative compared to a time period when no close relative died [16,17]. For index persons with more than one relative’s death during one year prior to the suicide, the most recent event was the one considered in the analyses. All time periods were analysed with regard to any close relative’s death, and stratified by the sex of the index person. For the analyses of the full year, the first and the second half-year and the first month, stratified analyses for age and separate analyses for each type of relationship (parent, sibling, child, and partner) were performed. Moreover, analyses related to death of a partner were stratified by sex and age of the index person.

All analyses were conducted using the statistical software IBM SPSS Statistics 22.

### Ethical considerations

The study population was based on linking several public national registers. Ethical vetting is always required when using register data in Sweden. The ethical vetting is performed by regional ethical review boards and the risk appraisal associated with the Law on Public Disclosure and Secrecy is done by data owners. The ethical review boards can however waive the requirement to consult the data subjects (or in case of minors/children the next of kin, careers or guardians) directly to obtain their informed consent, and will often do so if the research is supported by the ethical review board and the data has already been collected in some other context. Also, the institutional review board/ethics committee waived the need for written informed consent from the participants. Patient records/information was anonymized and deidentified prior to analysis by the authority, Statistics Sweden, which was responsible for data linkage. Researchers received de-identified data. According to these standards in Sweden this project has been evaluated and approved by the Regional Ethical Review Board of Karolinska Institutet, Stockholm, Sweden (case number 2010/1185-31/5).

## Results

The index persons comprised 21 721 men (69·9%) and 9 338 women (30·1%). The age at death ranged between 11 and 101 years (mean: 50·9, standard deviation: 18·8). In the case period (within the year before suicide), 1 223 index persons (3·9%) had experienced a death of a close relative. In the year before that (control period), 1 036 index persons (3·3%) were bereaved, resulting in an OR of 1·19 (95% CI 1·09–1·29). In total, 48 individuals had experienced deaths of close relatives during both years.

Table 1 shows that the ORs of suicide fluctuated during the different time periods within the first year after death of a close relative. The ORs of suicide were significantly increased during the first and the fifth month after bereavement, 1·77 and 1·70, respectively. The increased ORs in the first month were mainly confined to the first and fourth week, 3·43 and 2·17, respectively (Table 1). The OR for the first but not for the second half-year was significantly increased.

**Table 1:** Odds ratios (OR) and 95% Confidence Intervals (CI) of suicide within the first year after death of a close relative in 31 059 suicide victims in Sweden.

Time period	Case period, n^a^	Control period, n^a^	OR (95% CI)^b^
1^st^ week	48	14	3·43 (1·89–6·22)
2^nd^ week	27	22	1·23 (0·70–2·16)
3^rd^ week	35	25	1·40 (0·84–2·34)
4^th^ week	26	12	2·17 (1·09–4·29)
1^st^ month	140	79	1·77 (1·35–2·34)
2^nd^ month	103	100	1·03 (0·78–1·36)
3^rd^ month	110	99	1·11 (0·85–1·46)
4^th^ month	102	81	1·26 (0·94–1·69)
5^th^ month	119	70	1·70 (1·27–2·28)
6^th^ month	91	95	0·96 (0·72–1·28)
1^st^ half-year	659	519	1·27 (1·13–1·43)
7^th^ month	94	76	1·24 (0·91–1·67)
8^th^ month	101	79	1·28 (0·95–1·72)
9^th^ month	77	83	0·93 (0·68–1·27)
10^th^ month	98	81	1·21 (0·90–1·62)
11^th^ month	80	82	0·98 (0·72–1·33)
12^th^ month	90	88	1·02 (0·76–1·37)
2^nd^ half-year	529	478	1·11 (0·98–1·25)
1^st^ year	1175	988	1·19 (1·09–1·29)

^a^ Number of discordant exposed. Discordantly exposed index persons are differentially exposed in the case and control period. Concordantly exposed index persons (exposed in both case and control period) were uncommon and are not presented here.

^b^ The odds of being exposed to a close relative’s death within the year (i.e. 1–365 days) before the suicide (case periods) was compared with the odds of being exposed within the year before that (i.e. 366–730 days preceding the index person’s suicide, control periods).

These patterns were similar for women and men and across age groups in the stratified analyses (Tables 2 and 3). Table 4 shows the ORs of suicide within one month, the first and second half-year and one year following the death of a close relative, by type of relationship. Similar time patterns characterised by higher relative risks in the early period of bereavement were seen in all analyses. Estimates of suicide relative risk in bereaved partners were highest. Death of a partner resulted in a statistically significant increased relative risk of suicide in the first month (OR, 3·64; 95% CI, 2·02–6·58). Losing a child also comprised an increased OR of suicide in the first half-year following the death. Losing a sibling or a parent was not associated with a significantly increased risk of suicide in the year following the loss.

**Table 2:** Odds ratios (OR) and 95% Confidence Intervals (CI) of suicide after death of a close relative in 21 721 male and 9 338 female suicide victims in Sweden, stratified by sex.

	Men (n = 21 721)			Women (n = 9 338)		
Time period	Case period, n^a^	Control period, n^a^	OR (95% CI)^b^	Case period, n^a^	Control period, n^a^	OR (95% CI)^b^
1^st^ week	36	9	4·00 (1·93–8·30)	12	<7	2·40 (0·85–6·81)
2^nd^ week	19	17	1·12 (0·58–2·15)	8	<7	1·60 (0·52–4·89)
3^rd^ week	23	18	1·28 (0·69–2·37)	12	7	1·71 (0·68–4·35)
4^th^ week	19	9	2·11 (0·96–4·67)	7	<7	2·33 (0·60–9·02)
1^st^ month	99	56	1·77 (1·27–2·45)	41	23	1·78 (1·07–2·97)
1^st^ half-year	427	357	1·20 (1·04–1·38)	232	162	1·43 (1·17–1·75)
2^nd^ half-year	350	334	1·05 (0·90–1·22)	179	144	1·24 (1·00–1·55)
1^st^ year	767	686	1·12 (1·01–1·24)	408	302	1·35 (1·16–1·57)

^a^ Number of discordant exposed.

^b^ The odds of being exposed to a close relative’s death within the year (i.e. 1–365 days) before the suicide (case periods) was compared with the odds of being exposed within the year before that (i.e. 366–730 days preceding the index person’s suicide, control periods).

**Table 3:** Odds ratios (OR) and 95% Confidence Intervals (CI) of suicide after death of a close relative in 31 059 suicide victims in Sweden, stratified by age.

	Age range in years			
Time period	10–24, n = 2 847	25–44, n = 9 132	45–64, n = 11 287	65+, n = 7 793
	OR (95% CI)^a^	OR (95% CI)^a^	OR (95% CI)^a^	OR (95% CI)^a^
1^st^ month	1·00 (0·14–7·10)	1·93 (1·04–3·61)	1·52 (1·03–2·25)	2·25 (1·33–3·81)
1st half-year	0·75 (0·32–1·78)	1·28 (0·99–1·64)	1·22 (1·04–1·43)	1·43 (1·14–1·81)
2nd half-year	1·06 (0·54–2·10)	0·94 (0·72–1·22)	1·15 (0·97–1·36)	1·19 (0·92–1·55)
1st year	0·90 (0·53–1·52)	1·12 (0·93–1·34)	1·18 (1·05–1·33)	1·32 (1·11–1·57)

^a^ The odds of being exposed to a close relative’s death within the year (i.e. 1–365 days) before the suicide (case periods) was compared with the odds of being exposed within the year before that (i.e. 366–730 days preceding the index person’s suicide, control periods).

**Table 4:** Odds ratios (OR) and 95% Confidence Intervals (CI) of suicide after death of a close relative in suicide victims in Sweden, by type of relationship.

	Type of relationship			
Time period	Partner, n = 25 985^b^	Child, n = 31 059	Sibling, n = 24 452^c^	Parent, n = 24 452^c^
	OR (95% CI)^a^	OR (95% CI)^a^	OR (95% CI)^a^	OR (95% CI)^a^
1^st^ month	3·64 (2·02–6·58)	1·80 (0·60–5·37)	1·33 (0·63–2·82)	1·33 (0·92–1·94)
1^st^ half-year	1·79 (1·42–2·27)	1·73 (1·02–2·92)	1·14 (0·80–1·61)	1·10 (0·95–1·28)
2^nd^ half-year	1·21 (0·92–1·59)	1·07 (0·64–1·79)	1·14 (0·80–1·61)	1·08 (0·93–1·26)
1^st^ year	1·53 (1·28–1·82)	1·38 (0·96–1·99)	1·13 (0·89–1·45)	1·08 (0·98–1·21)

^a^ The odds of being exposed to a close relative’s death within the year (i.e. 1–365 days) before the suicide (case periods) was compared with the odds of being exposed within the year before that (i.e. 366–730 days preceding the index person’s suicide, control periods).

^b^ Only index persons who committed suicide in year 1993–2011 are included in the analysis, n = 25 985.

^c^ Only index persons born 1932 or later are included in the analysis, n = 24 452.

When the analyses of the relative risk of suicide within one month following the death of a partner were stratified by sex, a similar pattern was seen among men (OR, 3·50; 95% CI, 1·73–7·07) and women (OR, 4·00; 95% CI, 1·34–11·97) (data not shown in tables). Further stratifying these analyses by age, revealed that the ORs of suicide within one month after death of a partner were similar in the two oldest age groups. In the 45–64 age group, the OR was 4·33 (95% CI, 1·24–15·21) and in the 65+ age group, the OR was 3·46 (95% CI, 1·77–6·76) (data not shown in tables).

## Discussion

### Main findings

This large self-matched study of 31 059 suicides showed an increased relative risk of suicide after the death of a close relative within the first year after loss. The relative risk was highest in the first week and significantly increased in the first month and first six months. After the first half-year, no excess relative risk of suicide was found. Similar patterns in the association of bereavement and subsequent suicide with regard to sex and age were found. Death of a partner or child but not death of a sibling or parent was associated with a significantly increased relative risk of suicide. The strongest association in this study was found for death of a partner and subsequent suicide among index persons aged 45 and older in the first month of bereavement.

Our results are comparable to two previous studies reporting an excess suicide risk in the first month after loss of a partner or loss of a child [6, 9]. A study focusing on spousal bereavement also found an excess risk in the first week, in line with the present study [6]. Compared to these two earlier studies, the magnitude of the effect in our study was smaller. Potential explanations for this disparity include differences in the study population with regard to age as well as differences in control for confounders. The variations in suicide risk by time since the loss indicate certain sensitive time periods and may reflect individual variation in response to bereavement and in the suicidal process [18]. While the excess risk in the first week might be particularly related to impulsive suicidal behavior, the higher ORs in the fifth month might more reflect the emergence of depressive symptoms and related suicide risk [1,18].

Our result, of an increased relative risk of suicide after death of a partner, is in line with other studies focussing on slightly longer time frames, from six months up to two years [4,7,8]. In the present study, we could now show that this association was similar for women and men and was particularly strong in the first month after loss in index persons aged 45 or older. These findings provide important knowledge of a particularly sensitive time window for suicide after loss of a partner. Assessing possible suicide risk seems warranted in partners with established risk factors in the early period of bereavement.

The present study found a significantly increased relative risk of suicide within six months after loss of a child. A previous study reported a particularly high risk in the first month of bereavement [9]. The risk of mental ill-health following a child’s death has been found to vary depending on the cause of the child’s death, e.g. being accidental, suicidal, or natural [19,20]. While more knowledge is warranted with respect to potential differences in the grieving process related to different causes of the child’s death as well as the role of familiarly shared genetic susceptibility for suicidal behaviour, it is of note that the early period of bereavement implies a particular risk of suicide in bereaved parents.

We could not find any significant association between the death of a sibling or parent and subsequent suicide. These findings are contradictory to earlier studies [5,10]. These discrepancies can arise from differences in follow-up, design and in the age of the study population. Many of the previous studies that have investigated the risk of suicidal behaviour after death of a parent, focused on younger study populations [3,10,21–23]. It is possible that younger individuals are more sensitive to a parent’s death [23].

This study did not reveal any sex differences in the effect of bereavement on suicide risk. Earlier studies have reported sex differences in this association with regard to type of relationship [4,5,9]. Our study covered several types of relationships in the study population, which might explain these discrepancies. On the other hand, we did not find any sex differences with regard to death of a partner either. An earlier study has suggested that sex differences in the effect of death of a partner might depend on the studied age group [6]. Further studies are warranted to investigate if there are age differences and even differences in other characteristics, e.g. social support, in the way women and men respond to the loss of a partner. Due to the known sex differences in suicidal behaviour, studies investigating suicide attempt as outcome in addition to suicide might shed light on eventual differences with regard to sex and type of suicidal behaviour in response to bereavement.

### Strengths and limitations

This study used a case-crossover design. In this design, each individual is used as his or her own control which inherently controls for all time-stable confounding factors. Stable risk factors, such as for instance familial and/or genetic factors creating a susceptibility for suicide, may however be effect modifiers, which are important topics for future studies. The occurrence of time dependent factors, such as a life event related to the death of a relative, is possible, but unlikely to happen systematically. For this reason, it is unlikely to result in confounding.

A methodological assumption of the case-crossover design, which applies especially to our study, is that “If the exposure itself is not transient, one or more of its effects should be,…” [11]. In our case, the loss of a relative might imply a one-time uni-directional change in exposure status, but the effects on the risk of suicide appear to be transient. An illustration of this methodological assumption can be found in Mostofsky et. al’s. study on the effect of the death of a significant other on the risk of acute myocardial infarction [24].

We have chosen to use control information from one year earlier in the individual’s life, which adjusts for potential seasonal variation in the risk of suicide and overall mortality and complies with similar time spans used in previous studies. This control period was also chosen to minimize confounding from time-dependent factors. With the time period of one year, the impact from these factors is likely to be smaller than for periods with a larger time gap. On the other hand, choosing a case period of one year and a control period of one year before the case period might have led to underestimation of effects, as earlier studies have shown an increased suicide risk among the bereaved after one year [8,9]. Here it is worth mentioning that the index persons who were exposed both in the case and control periods did not contribute to the reported odds ratios. It is possible that repeated exposure to the deaths of relatives could create a cumulatively increased risk of suicide, which cannot be estimated in our present study.

Another major strength is the high quality and coverage of Swedish registers, which made it possible to include an exceptionally large study population containing all suicides with a known date of death, over a 22-years period. Despite the overall high quality of the registers, the coverage of the registers used for linking index persons to their close relatives varied across time [15]. The coverage in the Multi-Generation Register was lower for index persons born in the 1930s (30–73% linkage with biological mothers) than for individuals born in the 1990s (97–99% of coverage) [15]. This implied that linkage was not complete for some parts of the study population, which has led to missed exposure events. Still, since cases are used as their own controls, lack of linkage occurred to the same extent in both case and control periods and therefore has most probably not affected the validity of our results. This study examined the effect of bereavement related to a close relative. Bereavement due to the death of other relatives and close friends might also have a similar effect. Unfortunately, such significant others are difficult to identify through existing registers.
